# Phase I dose-escalation trial of AMXT 1501 dicaprate plus difluoromethylornithine: a dual-agent approach targeting immunosuppressive polyamine metabolism

**DOI:** 10.1016/j.esmoop.2025.105576

**Published:** 2025-09-06

**Authors:** S.A. Piha-Paul, A.W. Tolcher, A.L. Vandross, A.I. Spira, M.R. Burns

**Affiliations:** 1 The University of Texas MD Anderson Cancer Center, Houston, USA; 2 NEXT Oncology, San Antonio, USA; 3 NEXT Oncology, Austin, USA; 4 NEXT Oncology Virginia, Fairfax, USA; 5 Aminex Therapeutics, Inc., Kenmore, USA

**Keywords:** polyamine transporter, immunometabolism, ornithine decarboxylase, SLC3A2, ATP13A3, polyamine

## Abstract

**Background:**

Dysregulation of polyamine synthesis has been observed in various cancer cell types. A novel approach to depriving cancer cells of polyamines involves the use of difluoromethylornithine (DFMO) to block polyamine biosynthesis in combination with AMXT 1501, a potent inhibitor of polyamine transport. Preclinical mouse tumor models showed that the combination of AMXT 1501 plus DFMO had strong antitumor activity, together with evidence of a stimulated immune response against tumors.

**Materials and methods:**

This was a multicenter phase I, open-label, two-part dose-escalation study, with expansion, to evaluate the safety and preliminary efficacy of oral AMXT 1501 in combination with oral DFMO in patients with advanced solid tumors. In part 1, patients were treated with ascending doses of AMXT 1501 alone for 2 weeks and then in combination with DFMO for an additional 2 weeks. In part 2, AMXT 1501 was dosed with escalating DFMO doses to determine the recommended phase II dose (RP2D), with an expansion cohort to confirm the RP2D. Patients with unresectable, locally advanced, or metastatic solid tumors for which no standard therapy was recognized were eligible.

**Results:**

A total of 56 patients were enrolled and treated (20, 22, and 14 in part 1, part 2, and expansion, respectively). Patients were heavily pretreated; the median number of prior cancer treatments was 10.0. The most common treatment-emergent adverse events (TEAEs) were diarrhea (39.3%), nausea (37.5%), and vomiting (33.9%). There were no grade 4 or 5 TEAEs and no deaths due to TEAEs. Moderate antitumor activity was observed with 2 patients with confirmed responses and 16 patients with stable disease for an overall response rate of 6% and a clinical benefit rate of 49%.

**Conclusions:**

Overall, AMXT 1501 in combination with DFMO was safe and tolerated with evidence of preliminary clinical activity. The RP2D was determined to be AMXT 1501 600 mg twice daily plus DFMO 500 mg.

**Clinical trial identification:**

NCT03536728.

## Highlights

• First-in-human clinical testing of AMXT 1501 plus DFMO as a polyamine-targeting approach for therapy of solid tumors.
• Targeting tumor polyamine metabolism is a promising approach to reduce immunosuppression by these immunometabolites.
• Clinical testing of two agents targeting the same metabolite pathway presented challenges.
• PK and preliminary pharmacodynamic analyses support the possibility that target-engaging levels of drug agents were achieved.
• Clinical tolerability at the RP2D level was demonstrated in 24 patients.

## Introduction

Targeting the intrinsic and extrinsic metabolic pathways of tumor cells to facilitate a natural immune response is of interest in anticancer therapy. While considerable progress has been made in the field of cancer immunotherapy using large molecules against immune checkpoint inhibitors,^1^ not all patients benefit from this approach, and there remains an unmet medical need for new treatment options. Several small compounds targeting intracellular negative regulators of antitumor immune responses are in clinical development,^2^^,^^3^ and there exists significant potential for orthogonal immunometabolic approaches.

Polyamines are small organic polycations that are essential for DNA replication, translation, differentiation, and cell proliferation. Dysregulation of polyamine synthesis has been observed in various cancer cell types, and elevated levels of polyamines are associated with the progression of neuroblastoma, hepatocellular carcinoma, prostate, lung, breast, gastric, and colorectal cancers.^4^, ^5^, ^6^, ^7^ The biosynthesis of polyamines is directly induced by myelocytomatosis oncogene (MYC) amplification, driving downstream polyamine production.^8^^,^^9^ It is commonly thought that polyamines promote tumorigenesis by stimulating cell proliferation and angiogenesis^10^, ^11^, ^12^, ^13^, ^14^; however, polyamines may also exert immunosuppressive effects via multiple mechanisms to help tumors evade detection.^15^^,^^16^ Emerging data support the actions of the polyamine-dependent hypusination of eukaryotic initiation factor 5A as a key mechanism in this process.^17^, ^18^, ^19^, ^20^

There has been long-standing interest in targeting polyamines as a therapeutic approach for cancer,^21^^,^^22^ and studies using oral difluoromethylornithine (DFMO), which inhibits polyamine synthesis by irreversibly inhibiting ornithine decarboxylase (ODC), the rate-limiting enzyme in polyamine biosynthesis, have shown some success.^22^, ^23^, ^24^ Most recently, an analysis of patients with high-risk neuroblastoma (HRNB), who received up to 2 years of treatment with oral DFMO after completion of immunotherapy, showed significant improvement in both event-free survival and overall survival.^25^ The findings led to the recent approval by the Food and Drug Administration of oral DFMO (eflornithine, Iwilfin) to reduce the risk of relapse in adult and pediatric patients with HRNB.^26^ Separate studies have, however, revealed that DFMO inhibition of ODC leads to up-regulation of the polyamine transporter, resulting in increased uptake of polyamines derived from the diet and gut flora into the tumor cells.^27^^,^^28^ Thus, there may be benefit from inhibiting both polyamine biosynthesis and polyamine transport to truly starve a tumor of polyamines.^16^

A novel approach to depriving cancer cells of polyamines involves the use of DFMO to block polyamine biosynthesis in combination with AMXT 1501, an inhibitor of polyamine transport. AMXT 1501 is a polyamine mimetic that blocks cellular uptake of spermidine in the nanomolar range without crossing the cell membrane.^29^ No rescue from the growth inhibitory effects of DFMO occurred when AMXT 1501 was added to cells, which suggests that AMXT 1501 could not be taken up and metabolized to replenish required levels of cellular polyamines.^29^

Polyamine blocking therapy with DFMO in combination with AMXT 1501 has been shown to suppress tumor growth in multiple *in vivo* tumor models in mice.^16^^,^^29^, ^30^, ^31^, ^32^, ^33^ AMXT 1501 plus DFMO treatment caused very significant regressions in the majority (88%) of established carcinogen-induced squamous-cell carcinomas in ODC transgenic mice in which skin tumors were promoted by elevated epidermal ODC activity.^16^ AMXT 1501 plus DFMO produced an almost complete blockade of tumor growth, with a durable effect after treatment ceased. Evidence from this model suggested that an immune response could have been partially responsible for the robust efficacy results. In the most recent studies, AMXT 1501 plus DFMO showed pronounced anticancer activity in the transgenic mouse models of tyrosine hydroxylase MYC neuroblastoma (TH-MYCN)^32^ and diffuse intrinsic pontine glioma (DIPG).^33^ The finding that polyamine depletion therapy is an effective therapeutic approach leading to significant tumor growth delay in animal models prompted subsequent evaluation in clinical studies in patients with cancer.

AMXT 1501 alone and in combination with DFMO was evaluated in a first-in-human, phase I dose-escalation clinical trial testing the safety and tolerability in adult patients with solid malignancies.

## Materials and methods

### Study design

**Figure 1:**
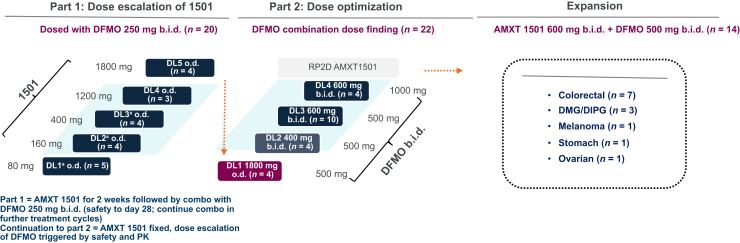
**Studydesign**. b.i.d., twice daily; DFMO, difluoromethylornithine; DIPG, diffuse intrinsic pontine glioma; DL, dose level; DMG, diffuse midline glioma; o.d., once daily; PK, pharmacokinetics. ^a^Required 14 day rest period between end of cycle 1 and start of cycle 2; removed at cohort 4 Part 1 as no safety issues.

This was a multicenter, phase I, open-label, two-part dose-escalation study, with an expansion cohort, to evaluate the safety and preliminary efficacy of oral AMXT 1501 in combination with oral DFMO in patients with advanced solid tumors (Figure 1). Dose escalation followed a 3 + 3 dose-escalation design.^34^

• In part 1, patients received ascending doses of AMXT 1501 dicaprate (hereafter referred to as AMXT 1501) alone [starting dose 80 mg (free base content) once daily (o.d.)], for 2 weeks, then in combination with DFMO 250 mg, dosed twice daily (b.i.d.) for an additional 2 weeks. Patients who successfully tolerated the drug combination for the 28-day treatment cycle could continue for multiple cycles.
• In part 2, AMXT 1501 was initially dosed at its final part 1 level [part 1 AMXT 1501 recommended phase II dose (RP2D), determined to be 1800 mg o.d.] with escalating DFMO dosing.
• An expansion cohort was included at the RP2D for both drugs to confirm the RP2D. The study was carried out in compliance with the International Council for Harmonisation Good Clinical Practices, including the ethical principles of the Declaration of Helsinki. All patients provided signed informed consent before any study-specific procedures were carried out.

### Investigational products

AMXT 1501 dicaprate (40 or 200 mg free base content) was administered in Vcaps Enteric™ capsules (Capsugel, Morristown, NJ). DFMO HCl H_2_O (250 or 500 mg salt weight content) was administered in hard gelatin capsules.

### Patients

Patients aged ≥18 years with Eastern Cooperative Oncology Group performance status (ECOG PS) 0-1 and unresectable, locally advanced, or metastatic solid tumors for which no standard therapy was recognized or for which standard therapy had failed were enrolled. Additional inclusion criteria included: histologically or cytologically documented disease [or radiologically documented disease in the case of DIPG or diffuse midline glioma (DMG)]; disease evaluable or measurable by Response Evaluable Criteria for Solid Tumors Version 1.1 (RECIST 1.1)^35^ or Response Assessment in Neuro-Oncology (RANO) for DIPG/DMG^36^; availability of tumor tissue or archival tissue or for expansion patients’ willingness to provide tumor biopsies; and adequate bone marrow and renal/hepatic function. Solid tumor patients with treated (surgically excised or irradiated) and stable brain metastases were eligible if the treatment was at least 4 weeks before initiation of study drug. Patients with stable brain metastases must not have required therapy with corticosteroids.

### Objectives

The primary objective of this study was to determine the safety and tolerability of oral AMXT 1501 in combination with DFMO in patients with advanced cancer. Secondary objectives included characterization of pharmacokinetics (PK) of AMXT 1501 and DFMO as well as evaluation of objective response rate (ORR) and duration of response (DOR). Exploratory objectives included pharmacodynamic assessment of the impact of AMXT 1501 on polyamine uptake by circulating lymphocytes.

### Dose escalation and determination of maximum tolerated dose

For determination of dose escalation, all toxicities, all available PK, and other relevant patient data occurring during the first cycle were considered. In addition, safety in patients ongoing in repeat cycles at lower doses was also taken into consideration by the Cohort Review Committee (CRC; consisting of investigators and the medical monitor), when making dose-escalation decisions. The determination of RP2D included the assessment of acute and chronic toxicities and tolerability (missing doses, dose reductions, etc.) and was informed by the experience of the totality of data. The maximum tolerated dose (MTD) was defined as the highest dose level at which one or fewer of six patients experienced dose-limiting toxicities (DLTs). All cycles were 4 weeks (28 days of treatment) and a rest/recovery period between cycles was only required if treatment-emergent toxicities were present and had not resolved to Common Terminology Criteria for Adverse Events (CTCAE) toxicity grade levels observed at baseline, or CTCAE grade (G) ≤1. A dose reduction at or after cycle 2 was granted in accordance with dose modification guidelines if, in the opinion of the investigator, a dose reduction was in the best interest of the patient.

### Study assessments

#### Safety

Safety was monitored throughout the study. The results of laboratory evaluations, audiograms (administered due to reports of hearing loss in prior clinical trials using DFMO^37^), vital signs, physical examinations, 12-lead electrocardiograms, and adverse events (AEs) were evaluated. All safety evaluations were carried out using the safety population, which included all patients who received at least one dose of study drug.

#### Efficacy

Efficacy assessments included determination of objective response, best overall response (BOR), DOR, and progression-free survival (PFS). The ORR and clinical benefit rate (CBR) were defined by the number and proportion of patients who achieved objective tumor response [complete response (CR) or partial response (PR) and CR + PR + stable disease (SD), respectively] using RECIST v1.1 or RANO and were assessed by the investigator. If SD was included in the calculation of CBR, SD status must be maintained for 16 weeks. Tumor assessments were carried out at screening, at the end of cycle 2 (i.e. week 8), and every two cycles thereafter. Efficacy analyses were conducted in the anticancer activity population, which included all patients who received at least one dose of study drug, had a baseline imaging scan, and at least one post-treatment scan.

#### Pharmacokinetics

The plasma PK samples were analyzed for AMXT 1501 and DFMO concentrations using a validated protein precipitation extraction procedure with liquid chromatography tandem mass spectrometry. The lower limit of quantitation (LLOQ) for AMXT 1501 in human plasma was 0.5 ng/ml. The LLOQ for DFMO in human plasma was 10 ng/ml. Reference standards for each analyte and labeled internal standards (AMXT 1501 ^13^C_4_ and DFMO-D_3_) were employed. Blood was obtained from all patients and collected in anticoagulant tubes (K2-EDTA). Plasma samples were stored at −80°C until analysis (both drugs were validated as stable under these storage conditions). The PK analysis population included all patients who received at least one dose of AMXT 1501 and had at least one quantifiable AMXT 1501 plasma concentration postdose.

#### AMXT 1501 target engagement

To evaluate the target engagement activity of AMXT 1501 after administration, an *ex vivo* pharmacodynamic assay assessing lymphocyte spermine uptake and inhibition of uptake by AMXT 1501 was developed.

The purpose of the whole blood assay is to quantify the inhibition of polyamine transport in T cells in human subjects receiving AMXT 1501. Whole blood collected from subjects receiving AMXT 1501 was diluted with serum-free media and stimulated with anti-CD3 and anti-CD28 to drive T-cell activation and proliferation for a total of 3 days. During the last 12-18 h of culture, a fluorescently labeled spermine analog (compound 5)^38^ was added to the *in vitro* culture to monitor polyamine uptake into cells. The functional activity (inhibitory effect) of AMXT 1501 on compound 5 uptake by T cells is then determined using flow cytometry by measuring the frequency and mean fluorescence intensity of compound 5+ T cells. Comparisons were made between the uptake in the pretreatment sample and samples taken over time postdose.

## Results

### Demographics and disposition

A total of 56 patients were enrolled and treated (20 in part 1, 22 in part 2, and 14 in expansion; Table 1). Overall (*n* = 56), the majority of patients were female (64%) and white or Caucasian (64%), with a median age of 58.5 years (range 20-78 years) (Table 2). All patients had a baseline ECOG PS of either 0 (7%) or 1 (93%) and all patients with baseline disease staging had either stage III (6%) or stage IV (89%) disease (baseline disease stage was unknown for three patients). Patients had received a median (standard deviation) of 10.0 (5.96) prior cancer treatments and had a median time since initial diagnosis of cancer to date of informed consent of 4.71 years. There was a wide range of tumor types (Table 2).

**Table 1:** Disposition and dose groups

Cohort	AMXT 1501	Part 1	Subjects, *n*
		DFMO	
1	80 mg o.d.	250 mg b.i.d.	5
2	160 mg o.d.	250 mg b.i.d.	4
3	400 mg o.d.	250 mg b.i.d.	4
4	1200 mg o.d.	250 mg b.i.d.	3
5^a^	1800 mg o.d.	250 mg b.i.d.	4
	Total		20

**Cohort**	**AMXT 1501**	**Part 2**	**Number of subjects**
		DFMO	
1	1800 mg o.d.	500 mg b.i.d.	4
2	400 mg b.i.d.^b^	500 mg b.i.d.	4
3^c^	600 mg b.i.d.^b^	500 mg b.i.d.	10
4	600 mg b.i.d.^b^	1000 mg b.i.d.	4
Expansion cohort^c^	600 mg b.i.d.^b^	500 mg b.i.d.	14
	Total	All patients	56

b.i.d., twice daily; DFMO, difluoromethylornithine; o.d., once daily; PK, pharmacokinetics; RP2D, recommended phase II dose.

a RP2D was AMXT 1501 1800 mg o.d.

b Evaluation of PK data suggested b.i.d. dosing would also provide more sustained 24-h drug exposure.

c RP2D was AMXT 1501 600 mg b.i.d. and DFMO 500 mg b.i.d.

**Table 2:** Patient demographics, other baseline characteristics, and disease history

	All patients
	AMXT 1501 80-1800 mg o.d.; 400 or 600 mg b.i.d.
	DFMO 500-1000 mg b.i.d.
	*N* = 56
Age,^a^ years	
Mean (standard deviation)	58.1 (14.14)
Median	58.5
Minimum-maximum	20-78
Age groups, *n* (%)	
<65 years	35 (63)
≥65 years	21 (38)
Sex, *n* (%)	
Male	20 (36)
Female	36 (64)
Ethnicity, *n* (%)	
Hispanic or Latino	11 (20)
Not Hispanic or Latino	42 (75)
Not reported	3 (5)
Race,^b^*n* (%)	
American Indian or Alaska Native	3 (5)
Asian	2 (4)
Black or African American	6 (11)
Native Hawaiian or Other Pacific Islander	1 (2)
White or Caucasian	36 (64)
Other	2 (4)
Not reported	7 (13)
Body mass index at baseline, kg/m^2^	
*n*	55
Mean (standard deviation)	28.65 (7.650)
Median	27.07
Minimum-maximum	18.3-53.2
ECOG PS at baseline,^c^*n* (%)	
0	4 (7)
1	52 (93)
Stage at baseline, *n* (%)	
IIIA	2 (4)
IIIB	1 (2)
IVA	29 (52)
IVB	21 (38)
Unknown	3 (5)
Time since initial diagnosis of cancer to date of informed consent, years^d^	
*n*	55
Mean (standard deviation)	6.23 (6.429)
Median	4.71
Minimum-maximum	0.3-30.5
Number of prior anticancer therapies	
Mean (standard deviation)	10.9 (5.96)
Median	10.0
Minimum-maximum	1-23
Tumor type, *n* (%)	
Adenocarcinoma of pancreas	1 (2)
Adenocarcinoma of rectosigmoid	1 (2)
Adenocarcinoma of rectosigmoid junction	1 (2)
Adenocarcinoma of stomach	1 (2)
Adenocarcinoma, NOS of transverse colon	1 (2)
Brainstem glioma	1 (2)
Breast cancer	1 (2)
Chondrosarcoma	1 (2)
Colon cancer	4 (7)
Colorectal adenocarcinoma	1 (2)
Colorectal carcinoma	1 (2)
Colorectal cancer	6 (11)
Diffuse midline glioma with h3k27m	1 (2)
Ductal breast cancer	1 (2)
Extraskeletal myxoid chondrosarcoma	1 (2)
Gastroesophageal junction adenocarcinoma to distant left supraclav	1 (2)
Glioblastoma multiforme of brain	1 (2)
Glioma	1 (2)
Jejunum/small bowel adenocarcinoma	1 (2)
Liposarcoma	1 (2)
Metastatic melanoma	1 (2)
Malignant epithelial tumor of ovary	1 (2)
Malignant melanoma of vulva	1 (2)
Malignant neoplasm of overlapping sites of cervix uteri	1 (2)
Mesothelioma	1 (2)
Metastatic adenocarcinoma of prostate	1 (2)
Metastatic breast cancer	1 (2)
Metastatic colorectal cancer	1 (2)
Metastatic leiomyosarcoma	1 (2)
Nasal angiosarcoma	1 (2)
Non-small-cell lung cancer	1 (2)
Ovarian cancer	3 (5)
Pancreatic adenocarcinoma	1 (2)
Pancreatic cancer	1 (2)
Peritoneal serous carcinoma	1 (2)
Primary malignant neoplasm of rectum	1 (2)
Prostate cancer	1 (2)
Rectal cancer	2 (4)
Sigmoid colon cancer	1 (2)
Squamous-cell carcinoma of cervix	1 (2)
Squamous-cell carcinoma of gum	1 (2)
Synovial sarcoma	1 (2)
Uterine cancer	1 (2)
Uterine sarcoma	1 (2)
Not reported	1 (2)

b.i.d., twice daily; DFMO, difluoromethylornithine; NOS, not otherwise specified; o.d., once daily.

a Age was calculated as the number of years between the date of birth and the date of signing the informed consent.

b More than one response could be provided for race and, as such, the percentage may total more than 100%.

c ECOG PS: Eastern Cooperative Oncology Group Performance Status: 0 = fully active; 1 = restricted in activity.

d Time since initial diagnosis of cancer to date of informed consent was calculated as the number of years between the date of initial diagnosis and the date of informed consent.

During part 1, a total of 20 patients received AMXT 1501 in combination with DFMO (250 mg b.i.d.) after 2 weeks of AMXT 1501 monotherapy (AMXT 1501 at doses of 80, 160, 400, 1200, and 1800 mg o.d.). The RP2D was 1800 mg o.d.

During part 2, a total of 22 patients received AMXT 1501 in combination with DFMO. In part 2, four patients in cohort 1 were dosed at AMXT 1800 mg o.d. plus DFMO 500 mg b.i.d.; however, following the occurrence of two DLTs including one patient with five or more missed doses of AMXT 1501 in cycle 1 due to related G1 nausea and one patient with G3 vomiting, the CRC recommended a lower daily dose of AMXT 1501 with b.i.d. dosing. Evaluation of PK data suggested that b.i.d. dosing would also provide more sustained 24-h drug exposure. A total of 4 patients in cohort 2 were dosed with AMXT 1501 400 mg b.i.d. plus DFMO 500 mg b.i.d.; 10 patients in cohort 3 were dosed with AMXT 1501 600 mg b.i.d. plus DFMO 500 mg b.i.d.; 4 patients in cohort 4 were dosed with AMXT 1501 600 mg b.i.d. plus DFMO 1000 mg b.i.d., but due to gastrointestinal (GI) intolerabilities (nausea, vomiting, diarrhea) observed in cohort 4, the cohort 3 dose was deemed the RP2D. In the expansion cohort, a total of 14 patients received AMXT 1501 in combination with DFMO at the RP2D. Therefore, a total of 24 patients received the RP2D (AMXT 1501 600 mg b.i.d. plus DFMO 500 mg b.i.d.).

Overall, 35 (63%) patients completed the first cycle of treatment. All patients discontinued treatment during the study; the most common primary reasons were disease progression (55%), followed by AEs (20%). The most common primary reasons for discontinuation from the study were death (38%), followed by withdrawal of consent (25%).

All 56 patients were included in the safety, PK, and pharmacodynamic populations. A total of 35 patients (63%) were included in the anticancer activity population.

### Safety

Overall, 38 patients (68%) had one or more treatment-related treatment-emergent adverse events (TEAEs) (Tables 3 and 4). The treatment-related TEAEs with the highest incidences (≥25 patients) were reported in the system organ class of GI disorders and included diarrhea (39%), nausea (38%), and vomiting (34%) (Tables 5 and 6). No other AMXT-related TEAE occurred in ≥10 patients. G3 treatment-related TEAEs were reported in 14 patients (25%). The only G3 treatment-related TEAEs occurring in more than one patient were nausea, vomiting, and diarrhea (11.5% each) and hypocalcemia (8.9%). No G4 or G5 treatment-related TEAEs were reported.

**Table 3:** Summary of overall and most common treatment-emergent AEs—part 1: ascending AMXT 1501 doses with fixed-dose DFMO (safety population)

Patients with, *n* (%)	Cohort 1	Cohort 2	Cohort 3	Cohort 4	Cohort 5	All part 1 patients
	AMXT 1501 80 mg o.d.	AMXT 1501 160 mg o.d.	AMXT 1501 400 mg o.d.	AMXT 1501 1200 mg o.d.	AMXT 1501 1800 mg o.d.	AMXT 1501 80-1800 mg o.d.
	DFMO 250 mg b.i.d.					
	*n* = 5	*n* = 4	*n* = 4	*n* = 3	*n* = 4	*n* = 20
TEAEs^a^	5 (100)	4 (100)	3 (75)	3 (100)	4 (100)	19 (95)
DLTs^b^	0	0	0	0	0	0
TEAEs during first 14 days of cycle 1	5 (100)	3 (75)	3 (75)	3 (100)	4 (100)	18 (90)
Treatment-related TEAEs during first 14 days of cycle 1	5 (100)	2 (50)	3 (75)	3 (100)	3 (75)	16 (80)
Serious TEAEs	2 (40)	0	2 (50)	2 (67)	1 (25)	7 (35)
Treatment-related serious TEAEs	0	0	0	0	0	0
AMXT 1501-related TEAEs	5 (100)	2 (50)	3 (75)	3 (100)	4 (100)	17 (85)
DFMO-related TEAEs	3 (60)	2 (50)	3 (75)	3 (100)	3 (75)	14 (70)
TEAEs related to either AMXT 1501 or DFMO	5 (100)	2 (50)	3 (75)	3 (100)	4 (100)	17 (85)
TEAEs related to both AMXT 1501 and DFMO	2 (40)	2 (50)	3 (75)	3 (100)	3 (75)	13 (65)
Grade 3 TEAEs^c^	1 (20)	1 (25)	0	1 (33)	2 (50)	5 (25)
Grade 4 TEAEs^c^	0	0	0	0	0	0
Grade 5 TEAEs^c^	2 (40)	0	2 (50)	1 (33)	1 (25)	6 (30)
Treatment-related grade 3 TEAEs^c^	1 (20)	0	0	2 (67)	0	3 (15)
Treatment-related grade 4 and/or grade 5 TEAEs^c^	0	0	0	0	0	0
TEAEs leading to AMXT 1501 discontinuation	2 (40)	0	2 (50)	2 (67)	0	6 (30)
TEAEs leading to DFMO discontinuation	1 (20)	0	2 (50)	2 (67)	0	5 (25)
TEAEs leading to AMXT 1501 dose interruption	2 (40)	2 (50)	0	0	0	4 (20)
TEAEs leading to DFMO dose interruption	2 (40)	2 (50)	1 (25)	0	0	5 (25)
TEAEs with fatal outcome	2 (40)	0	2 (50)	1 (33)	1 (25)	6 (30)

Percentages are based on total number of patients (*n*) in each column.

AE, adverse event; AMXT 1501, AMXT 1501 dicaprate; CTCAE, Common Terminology Criteria for Adverse Events; DLT, dose-limiting toxicity; DFMO, difluoromethylornithine; NCI, National Cancer Institute; TEAE, treatment-emergent adverse event.

a Treatment-emergent AE was defined as any new AE that began, or any pre-existing condition that worsened in severity after one or more doses of study treatment were administered and throughout the treatment period until 30 days after stopping of study treatment. Treatment-related TEAEs included those with a missing or unknown drug relationship or a drug relationship of ‘definitely related’ or ‘possibly related’.

b Used the DLT population, which included patients who completed cycle 1 treatment DLT observation period without missing five or more doses of AMXT 1501 and/or DFMO. The number of patients in the DLT population was the denominator for reporting percent.

c Toxicities were graded using the NCI-CTCAE version 5. Patients with multiple events were counted once under the worst toxicity/severity.

**Table 4:** Summary of overall and most common treatment-emergent AEs—part 2: AMXT 1501 doses with ascending DFMO doses and expansion (safety population)

Patients with, *n* (%)	Cohort 1	Cohort 2	Cohort 3	Cohort 4	All part 2 patients	Expansion cohort	All patients
	AMXT 1501 1800 mg o.d.	AMXT 1501 400 mg b.i.d.	AMXT 1501 600 mg b.i.d.	AMXT 1501 600 mg b.i.d.	AMXT 1501 400 mg b.i.d. to 1800 mg o.d.	AMXT 1501 600 mg b.i.d.	AMXT 1501 400 mg b.i.d. to 1800 mg o.d.
	DFMO 500 mg b.i.d.	DFMO 500 mg b.i.d.	DFMO 500 mg b.i.d.	DFMO 1000 mg b.i.d.	DFMO 500-1000 mg b.i.d.	DFMO 500 mg b.i.d.	DFMO 500-1000 mg b.i.d.
	*n* = 4	*n* = 4	*n* = 10	*n* = 4	*n* = 22	*n* = 14	*n* = 56
TEAEs^a^	4 (100)	4 (100)	10 (100)	4 (100)	22 (100)	14 (100)	55 (98)
DLTs^b^	2/4 (50)	0/3 (0)	1/7 (14)	3/4 (75)	6/18 (33)	0	6/35 (17)
TEAEs during first 14 days of cycle 1	4 (100)	3 (75)	10 (100)	4 (100)	21 (95)	13 (93)	52 (93)
Treatment-related TEAEs during first 14 days of cycle 1	3 (75)	1 (25)	5 (50)	3 (75)	12 (55)	10 (71)	38 (68)
Serious TEAEs	2 (50)	3 (75)	5 (50)	2 (50)	12 (55)	10 (71)	29 (52)
Treatment-related serious TEAEs	1 (25)	0 (0)	2 (20)	1 (25)	4 (18)	4 (29)	8 (14)
AMXT 1501-related TEAEs	3 (75)	2 (50)	7 (70)	3 (75)	15 (68)	10 (71)	42 (75)
DFMO-related TEAEs	3 (75)	2 (50)	7 (70)	3 (75)	15 (68)	10 (71)	39 (70)
TEAEs related to either AMXT 1501 or DFMO	3 (75)	2 (50)	7 (70)	3 (75)	15 (68)	10 (71)	42 (75)
TEAEs related to both AMXT 1501 and DFMO	3 (75)	2 (50)	7 (70)	3 (75)	15 (68)	10 (71)	38 (68)
Grade 3 TEAEs^c^	2 (50)	1 (25)	5 (50)	0	8 (36)	9 (64)	22 (39)
Grade 4 TEAEs^c^	0	0	1 (10)	0	1 (5)	0	1 (2)
Grade 5 TEAEs^c^	1 (25)	3 (75)	1 (10)	2 (50)	7 (32)	2 (14)	15 (27)
Treatment-related grade 3 TEAEs^c^	3 (75)	0 (0)	2 (20)	1 (25)	6 (27)	5 (36)	14 (25)
Treatment-related grade 4 and/or grade 5 TEAEs^c^	0	0	0	0	0	0	0
TEAEs leading to AMXT 1501 discontinuation	3 (75)	0	2 (20)	3 (75)	8 (36)	5 (36)	19 (34)
TEAEs leading to DFMO discontinuation	3 (75)	0	2 (20)	3 (75)	8 (36)	5 (36)	18 (32)
TEAEs leading to AMXT 1501 dose interruption	3 (75)	2 (50)	6 (60)	1 (25)	12 (55)	11 (79)	27 (48)
TEAEs leading to DFMO dose interruption	3 (75)	2 (50)	6 (60)	0	11 (50)	11 (79)	27 (48)
TEAEs with fatal outcome	1 (25)	3 (75)	1 (10)	2 (50)	7 (32)	2 (14)	15 (27)

Percentages are based on total number of patients (*n*) in each column.

AE, adverse event; AMXT 1501, AMXT 1501 dicaprate; b.i.d., twice daily; CTCAE, Common Terminology Criteria for Adverse Events; DFMO, difluoromethylornithine; DLT, dose-limiting toxicity; *n*, number of patients with data; NCI, National Cancer Institute; o.d., once daily; TEAE, treatment-emergent adverse event.

a Treatment-emergent AE was defined as any new AE that began, or any pre-existing condition that worsened in severity after one or more doses of study treatment were administered and throughout the treatment period until 30 days after stopping of study treatment. Treatment-related TEAEs included those with a missing or unknown drug relationship or a drug relationship of ‘definitely related’ or ‘possibly related’.

b Used the DLT population, which included patients who completed cycle 1 treatment DLT observation period without missing five or more doses of AMXT 1501 and/or DFMO. The number of patients in the DLT population was the denominator for reporting percent.

c Toxicities were graded using the NCI-CTCAE version 5. Patients with multiple events were counted once under the worst toxicity/severity.

**Table 5:** Summary of most common (>2 patients overall) treatment-related treatment-emergent AEs and grade ≥3 treatment-related treatment-emergent AEs—part 1: ascending AMXT 1501 doses with fixed-dose DFMO (safety population)

Patients with, *n* (%)	Cohort 1	Cohort 2	Cohort 3	Cohort 4	Cohort 5	All part 1 patients
	AMXT 1501 80 mg o.d.	AMXT 1501 160 mg o.d.	AMXT 1501 400 mg o.d.	AMXT 1501 1200 mg o.d.	AMXT 1501 1800 mg o.d.	AMXT 1501 80-1800 mg o.d.
	DFMO 250 mg b.i.d.					
	*n* = 5	*n* = 4	*n* = 4	*n* = 3	*n* = 4	*n* = 20
≥1 treatment-related treatment-emergent AE^a^	5 (100)	2 (50)	3 (75)	3 (100)	4 (100)	17 (85)
Gastrointestinal disorders	3 (60)	2 (50)	3 (75)	3 (100)	4 (100)	15 (75)
Nausea	2	1	1	3	2	9
Diarrhea	1	1	1	2	2	7
Vomiting	0	0	2	2	3	7
General disorders and administrative site condition	4 (80)	1 (25)	1 (25)	0	1 (25)	7 (35)
Pyrexia	3	1	0	0	1	5
Fatigue	2	0	1	0	1	4
Metabolism and nutrition disorders	1 (20)	0	1 (25)	0	1 (25)	3 (15)
Decreased appetite	1	0	1	0	0	2
Blood and lymphatic system disorders	0	1 (25)	0	1 (33)	0	2 (10)
Anemia	0	1	0	1	0	2
≥1 grade ≥3 treatment-related treatment-emergent AE^b^	1 (20)	0	0	2 (67)	0	3 (15)
Gastrointestinal disorders	0	0	0	1 (33)	0	1 (5)
Diarrhea	0	0	0	1	0	1
Blood and lymphatic system disorders	0	0	0	1 (33)	0	1 (5)
Anemia	0	0	0	1	0	1
Musculoskeletal and connective tissue disorders	1 (20)	0	0	0	0	1 (5)
Arthritis	1	0	0	0	0	1

Percentages are based on total number of patients (*n*) in each column.

AE, adverse event; AMXT 1501, AMXT 1501 dicaprate; CTCAE, Common Terminology Criteria for Adverse Events; DFMO, difluoromethylornithine; DLT, dose-limiting toxicity; NCI, National Cancer Institute; TEAE, treatment-emergent adverse event.

a Treatment-emergent AE was defined as any new AE that began, or any pre-existing condition that worsened in severity after one or more doses of study treatment were administered and throughout the treatment period until 30 days after stopping of study treatment. Treatment-related TEAEs included those with a missing or unknown drug relationship or a drug relationship of ‘definitely related’ or ‘possibly related’.

b Toxicities were graded using the NCI-CTCAE version 5. Patients with multiple events were counted once under the worst toxicity/severity.

**Table 6:** Summary of most common (>2 patients overall) treatment-related treatment-emergent AEs and grade ≥3 treatment-related treatment-emergent AEs—part 2: AMXT 1501 doses with ascending DFMO doses and expansion (safety population)

Patients with, *n* (%)	Cohort 1	Cohort 2	Cohort 3	Cohort 4	All part 2 patients	Expansion cohort	All patients
	AMXT 1501 1800 mg o.d.	AMXT 1501 400 mg b.i.d.	AMXT 1501 600 mg b.i.d.	AMXT 1501 600 mg b.i.d.	AMXT 1501 400 mg b.i.d. to 1800 mg o.d.	AMXT 1501 600 mg b.i.d.	AMXT 1501 400 mg b.i.d. to 1800 mg o.d.
	DFMO 500 mg b.i.d.	DFMO 500 mg b.i.d.	DFMO 500 mg b.i.d.	DFMO 1000 mg b.i.d.	DFMO 500-1000 mg b.i.d.	DFMO 500 mg b.i.d.	DFMO 500-1000 mg b.i.d.
	*n* = 4	*n* = 4	*n* = 10	*n* = 4	*n* = 22	*n* = 14	*n* = 56
≥1 treatment-related treatment-emergent AE^a^	3 (75)	2 (50)	7 (70)	3 (75)	15 (68)	10 (71)	42 (75)
Gastrointestinal disorders	3 (75)	1 (25)	7 (70)	3 (75)	14 (64)	10 (71)	39 (70)
Diarrhea	2	1	5	2	10	9	26
Nausea	1	0	5	3	9	8	26
Vomiting	2	0	5	3	10	9	26
General disorders and administrative site conditions	0	0	2 (20)	1 (25)	3 (14)	2 (14)	12 (21)
Fatigue	0	0	1	1	2	2	8
Pyrexia	0	0	0	0	0	0	5
Metabolism and nutrition disorders	1 (25)	0	1 (10)	0	2 (9)	3 (21)	8 (14)
Hypocalcemia	0	0	1	0	1	2	3
Blood and lymphatic system disorders	0	1 (25)	1 (10)	1 (25)	3 (14)	0	5 (9)
Anemia	0	0	1	1	2	0	4
Nervous system disorders	2 (50)	0	2 (20)	0	4 (18)	1 (7)	5 (9)
Dizziness	1	0	2	0	3	0	3
≥1 grade ≥3 treatment-emergent AE^b^	3 (75)	0	2 (20)	1 (25)	6 (27)	5 (36)	14 (25)
Gastrointestinal disorders	3 (75)	0	1 (10)	1 (25)	5 (23)	4 (29)	10 (18)
Nausea	1	0	1	1	3	3	6
Vomiting	1	0	1	1	3	3	6
Diarrhea	1	0	0	1	2	3	6
Constipation	0	0	0	0	0	1	1
General disorders and administrative site conditions	0	0	0	0	0	1 (7)	1 (2)
Fatigue	0	0	0	0	0	1	1
Metabolism and nutrition disorders	0	0	1 (10)	0	1 (5)	2 (14)	3 (5)
Hypocalcemia	0	0	3	0	3	2	5
Investigations	1 (25)	0	0	0	1 (5)	0	1 (2)
Alanine aminotransferase increased	1	0	0	0	1	0	1
Aspartate aminotransferase increased	1	0	0	0	1	0	1
Skin and subcutaneous tissue disorders	0	0	0	0	0	1 (7)	1 (2)
Rash	0	0	0	0	0	1	1

Percentages are based on total number of patients (*N*) in each column.

AE, adverse event; AMXT 1501, AMXT 1501 dicaprate; b.i.d., twice daily; CTCAE, Common Terminology Criteria for Adverse Events; DFMO, difluoromethylornithine; DLT, dose-limiting toxicity; NCI, National Cancer Institute; o.d., once daily; TEAE, treatment-emergent adverse event.

a Treatment-emergent AE was defined as any new AE that began, or any pre-existing condition that worsened in severity after one or more doses of study treatment were administered and throughout the treatment period until 30 days after stopping of study treatment. Treatment-related TEAEs included those with a missing or unknown drug relationship or a drug relationship of ‘definitely related’ or ‘possibly related’.

b Toxicities were graded using the NCI-CTCAE version 5. Patients with multiple events were counted once under the worst toxicity/severity.

There was no strong evidence for any AMXT 1501 dose relationship with the proportions of patients reported with TEAEs, treatment-related TEAEs or serious AEs (SAEs), TEAEs leading to either AMXT 1501 or DFMO discontinuation, or TEAEs leading to dose interruption. The most common (≥25 patients) TEAEs included diarrhea (64.3%), nausea (57.1%), vomiting (53.6%), and fatigue (44.6%).

Overall, no patients in part 1 and eight patients in part 2 and expansion had treatment-related SAEs, including vomiting (five patients), nausea (four patients), hypocalcemia (three patients), diarrhea (two patients), and fatigue (one patient).

Overall, TEAEs leading to permanent AMXT 1501 discontinuation occurred in 19 patients (34%); the most common were vomiting (4 patients), nausea (3 patients), and hypocalcemia (2 patients). Overall, 27 patients (48%) had TEAEs leading to AMXT 1501 treatment interruption; the most common were vomiting (9 patients), nausea (7 patients), and diarrhea (5 patients). As the trial progressed, patients received prophylactic antiemetics (prochlorperazine, ondansetron, dronabinol, promethazine, palonosetron, hyoscine), which greatly reduced the incidence of these GI AEs.

### Efficacy

**Figure 2:**
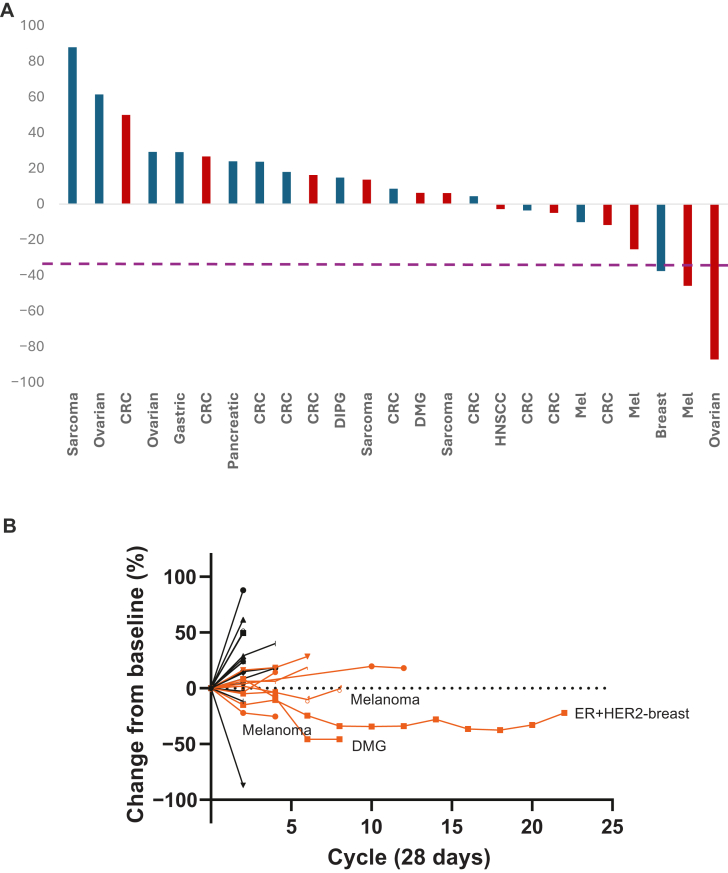
**Efficacy results.** (A) Waterfall plot (anticancer activity population). Patients shown in red received the recommended phase II dose (RP2D; AMXT 1501 600 mg b.i.d. with DFMO 500 mg b.i.d.). (B) Spider plot (anticancer activity population). Patients who received more than three cycles are noted in orange. CRC, colorectal cancer; DMG, diffuse midline glioma; ER, estrogen receptor; HER2, human epidermal growth factor receptor 2; HNSCC, head and neck squamous-cell carcinoma; Mel, melanoma.

Overall, two confirmed responses were reported in the anticancer activity population (*n* = 35), for an ORR of 6% [95% confidence interval (CI) 0.70% to 19.16%]. An additional 16 patients (46%) had a BOR of SD, for a CBR of 49% (95% CI 31.38% to 66.01%). Both confirmed PRs were in cohort 3 of part 2 at the RP2D (AMXT 1501 600 mg b.i.d., DFMO 500 mg b.i.d.). The two responses took time to develop, with one PR first reported on study day 108 and the other PR first reported on study day 231. The PR on study day 108 was reported in a 21-year-old male patient with DMG and was maintained for 1.8 months, while the PR on study day 231 was reported in a 73-year-old female patient with breast cancer and was maintained for 13 months. Of note, one patient with glioma was reported with a BOR of SD; however, the SD was not maintained for ≥16 weeks, and thus according to protocol, the patient was not included in the CBR calculation. Response data are shown in Figure 2. An overall trend for better response was observed for patients treated for more than three cycles.

The Kaplan–Meier estimated median DOR was not calculable with only two responses, one of which was ongoing (censored at 1.8 months’ duration) and the other with a PD after 13.0 months’ duration. Overall, the Kaplan–Meier estimated median PFS time was 2.3 months (95% CI 1.84-5.39 months), with estimated PFS probabilities at 3, 6, 9, and 12 months of 43%, 23%, 15%, and 15%, respectively.

### Pharmacokinetics

**Figure 3:**
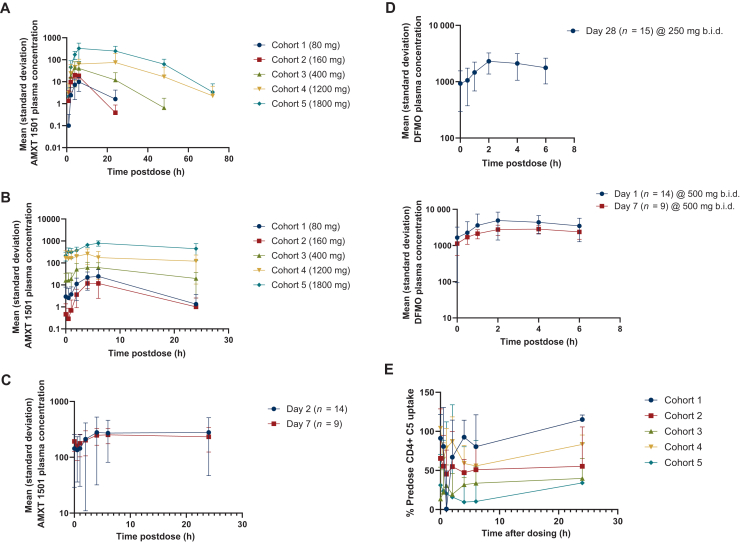
**Pharmacokinetics of AMXT 1501.** (A) AMXT 1501 mean concentration versus time by dose—part 1, cycle 1, day 1, administered as monotherapy with o.d. dosing. (B) AMXT 1501 mean concentration versus time by dose—part 1, cycle 1, day 14, administered as monotherapy with o.d. dosing. (C) AMXT 1501 600 mg b.i.d. (1200 mg total dose) mean concentration versus time—expansion cohort, cycle 1, day 2 and 7, administered in combination with DFMO 500 mg b.i.d. (D) DFMO PK profiles for all part 1 patients treated with 250 mg DFMO b.i.d. (in combination with various AMXT 1501 dicaprate dose levels) (upper), or 500 mg DFMO b.i.d. in expansion patients (lower). (E) Pharmacodynamic AMXT 1501 inhibition of fluorescently labeled polyamine uptake in part 1 patients after day 14 of AMXT 1501-alone therapy. AMXT 1501, AMXT 1501 dicaprate; b.i.d., twice daily; DFMO, difluoromethylornithine; o.d., once daily; PK, pharmacokinetics.

The PK of AMXT 1501 is shown in Figure 3. AMXT 1501 exposure based on mean maximum plasma concentration (*C*_max_) increased in a near dose-proportional manner over the 80-1200 mg dose range. AMXT 1501 steady state appeared to be obtained by cycle 1 day 14. After repeat dosing, the amount of accumulation of AMXT 1501 was highly variable between subjects, with some subjects showing little or no accumulation and others demonstrating accumulation of up to 10-fold. There was no consistent effect of DFMO administration on AMXT 1501 exposure.

The PK of DFMO 250 mg or 500 mg b.i.d. is shown in Figure 3D. DFMO exposure increased in an approximately dose-proportional manner between the 500 and 1000 mg/day dose levels and was variable between patients. There was no apparent effect of AMXT 1501 dose level on DFMO exposure and no substantial change in DFMO exposure with repeat dosing.

Interestingly, measurement of AMXT 1501 levels in a tumor biopsy collected after 30 days of treatment in a melanoma patient showed strikingly high levels of drug (3480 ng/ml). This patient was treated at the expansion RP2D level and showed a predose AMXT 1501 plasma concentration on day 8 of cycle 1 of 296 ng/ml, reflecting a 12-fold tumor-to-plasma ratio.

#### Pharmacodynamics—blood-based AMXT 1501 target engagement

An *ex vivo* blood-based pharmacodynamic assay was developed to measure the inhibition of uptake of a fluorescently labeled polyamine analog by AMXT 1501 into activated CD4+ lymphocytes in culture. Using a fluorescence-activating cell sorting system, tracking of the cellular uptake of the fluorescence of this labeled polyamine was followed after CD3/CD28 activation. In whole human blood samples treated with 0-2000 nM AMXT 1501, maximal fluorescent-labeled spermine uptake inhibition in the absence of DFMO occurred at 250 nM AMXT 1501 with a half maximum inhibitory concentration (ED_50_) of ∼75 nM. As expected, the addition of DFMO to the culture increased the uptake of the fluorescent marker, and AMXT 1501 was still able to potently inhibit this transport. Comparisons made between uptake in the pretreatment sample and samples taken over time following dosing show sustained AMXT 1501 target engagement activity. Figure 3E shows data from part 1 patients measured at day 14 of AMXT 1501-alone repeat dosing without DFMO co-therapy, showing AMXT 1501 dose response with increasing label uptake inhibition with more durable inhibition at higher dose levels. Importantly, the breast cancer patient with a PR showed near-complete inhibition when measured after day 7 of AMXT 1501 plus DFMO treatment. These data support a dose-dependent and sustained clinical target engagement following oral delivery of AMXT 1501 to cancer patients but correlation to response must await further data.

## Discussion

This was a first-in-human phase I study of the polyamine transport inhibitor, AMXT 1501, plus the polyamine synthesis inhibitor DFMO in heavily pretreated (median of 10.0 prior cancer treatments) adult patients with solid malignancies. Treatment with the combination was safe and well tolerated up to a dose of AMXT 1501 1800 mg o.d. plus DFMO 250 mg b.i.d. in part 1 of dose escalation. Clinical testing of two agents targeting the same metabolic pathway presented challenges, which were overcome with a trial design involving two parts evaluating AMXT 1501 monotherapy for the first 2 weeks and subsequent combination therapy with low-dose DFMO in part 1, followed by dose escalation of DFMO in part 2. These were followed by a dose-expansion phase. The clinical dose escalation in part 1 identified an MTD for AMXT 1501 1800 mg o.d. when given with DFMO 250 mg b.i.d.; however, in part 2, AMXT 1501 dosing was amended to b.i.d. following DLTs when administered in combination with DFMO 500 mg b.i.d. Based on the PK, pharmacodynamic, and tolerability data, the RP2D selected for evaluation in the dose-expansion phase was AMXT 1501 600 mg b.i.d. in combination with DFMO 500 mg b.i.d. Moderate antitumor activity was observed with two confirmed PRs and an additional 16 patients with SD for an ORR and a CBR of 6% and 49%, respectively.

The assessment of target engagement using the *ex vivo* blood-based pharmacodynamic assay showed sustained AMXT 1501 target engagement activity. While the assessments do not directly demonstrate AMXT 1501 target engagement in patients’ tumors, they show that AMXT 1501 present in patient blood samples after per os dosing is active and inhibits T-cell uptake *ex vivo*. These pharmacodynamic assays demonstrate that AMXT 1501 in circulation is functional, with clinical AMXT 1501 PK concentrations consistent with those shown to be efficacious in mouse cancer models.

Nonclinical data helped inform the expected clinical target engagement concentrations. The predicted effective dose 95% (ED_95_) value of AMXT 1501 is 100 nM in combination with DFMO against tumor cells in culture. AMXT 1501 plus DFMO displays potent cytostatic effects against multiple tumor cell lines, with an average half maximal effective concentration (EC_50_) value of 51 nM [melanoma (A375, 10 nM); breast (MDA-MB-231, 31 nM); prostate (PC-3, 44 nM); and ovarian (SK-OV-3, 120 nM)].^16^ These plasma AMXT 1501 levels were achieved clinically in patients treated at 600 mg AMXT 1501 given b.i.d. (RP2D) or higher dosages. Plasma drug levels are a surrogate of tumor levels, and nonclinical evidence from tumor-bearing mice suggests a high tumor association of AMXT 1501. This was supported by measurement of high levels of AMXT 1501 in a melanoma tumor biopsy [3480 ng/ml (6.12 μM)] obtained after 30 days of treatment of a patient in the dose-expansion phase at the RP2D level. This patient’s predose AMXT 1501 plasma concentration on day 8 of cycle 1 was 296 ng/ml, reflecting a 12-fold tumor-to-plasma drug ratio.

The predicted ED_95_ concentration of DFMO is 100 μM or 18 200 ng/ml, and oral delivery in combination with AMXT 1501 was unable to achieve this level. Given the lack of tumor biopsies collected, DFMO’s engagement of its ODC target was unable to be measured. Other recent studies using DFMO for neuroblastoma treatment in combination with chemotherapy looked at the PK through levels and observed 65.8 μM (95% CI 40.6-106.7 μM) in children treated at their RP2D level of 6750 mg/m^2^/day split three times a day.^39^ Given that GI toxicities were dose limiting with oral delivery, achieving these plasma levels of DFMO when combined with oral AMXT 1501 will be challenging without antiemetic prophylaxis, which were introduced as this trial progressed and reduced the incidence of GI toxicities. In future clinical studies, there would be a recommended antiemetic prophylaxis regimen.

The molecular identification of the mammalian polyamine transporter has seen recent progress. Gamble et al. reported that solute carrier family 3 member 2 (SLC3A2) was the key transporter involved in polyamine uptake in neuroblastoma cells.^32^ An additional candidate protein, ATP13A3, a P-type ATPase has been reported to be involved in polyamine uptake.^40^, ^41^, ^42^ Recently reported fluorescent sensors to measure subcellular polyamine concentrations have great appeal for further exploring the cellular biology of polyamines.^43^^,^^44^

Interestingly, only upon progressing to immunocompetent mouse tumor models was the pronounced immunological action of dual polyamine uptake and biosynthesis inhibition observed.^16^ Tumor regressions were observed, and measurements of immune system tumor engagement were demonstrated. Increased tumor-associated lymphocytes were noted, together with a significant reduction in the number of immunosuppressive myeloid-deprived suppressor cells in the tumors. Treatment of cancer cells in culture with AMXT 1501 plus DFMO induces a cytostatic not cytotoxic cellular response. It is important to note that the two PRs did take time to develop, implying that a delay for immune system activation is involved. The implications of this timeframe of response and the potential for combining AMXT 1501 plus DFMO with other agents will be addressed in future trials.
